# Characterizing the transmission dynamics of *Trypanosoma cruzi* in *Triatoma sanguisuga* collected from dog kennels in southern Texas

**DOI:** 10.1186/s13071-025-06917-6

**Published:** 2025-07-15

**Authors:** Carolina Hernandez, Roy Madigan, Weimar D. Briñez, Alberto Paniz-Mondolfi, Juan David Ramírez

**Affiliations:** 1 Molecular Microbiology Laboratory, Department of Pathology, Molecular and Cell-Based Medicine, Icahn School of Medicine at Mount Sinai, New York, NY USA; 2 Animal Hospital of Smithson Valley, 286 Singing Oaks, Ste 113, Spring Branch, TX 78070 USA; 3 Centro de Investigaciones en Microbiología y Biotecnología-UR (CIMBIUR), School of Sciences and Engineering, Universidad del Rosario, Bogota, Colombia

**Keywords:** Chagas disease, Vector-borne diseases, Parasites, Zoonoses, *Trypanosoma*, Insect vectors, Public health, Genotype, Population, Rural

## Abstract

**Background:**

Chagas disease, caused by the protozoan *Trypanosoma cruzi*, remains a significant public health issue in South America, with increasing concern over its potential transmission in the USA. *Triatoma sanguisuga*, a triatomine vector, is found in Southern states of the USA, including Texas, raising questions about the local transmission dynamics of *T. cruzi*. This study aims to characterize *Trypanosoma cruzi* infection in *Triatoma sanguisuga* collected from dog kennels in Bulverde and Spring Branch, Texas, with a focus on parasite prevalence and load, genotypic diversity, and blood-feeding sources.

**Methods:**

A total of 48 *T. sanguisuga* insects were collected from kennels in Bulverde (*N* = 37) and Spring Branch (*N* = 11). DNA extraction was followed by quantitative polymerase chain reaction (qPCR) to detect and quantify *T. cruzi*, genotyping via Oxford Nanopore Sequencing of the Miniexon gene, and blood-feeding source identification using the 12S rRNA gene was also conducted. Statistical analysis was performed to assess differences in parasitic load among the locations.

**Results:**

Of the 48 insects, 81.1% from Bulverde and 100% from Spring Branch tested positive for *T. cruzi*. The median parasitic load was log_10_ 8.09 equivalent parasites/mL, with significant differences in parasitic load between locations. Genotyping revealed that all samples were infected with TcI, with some co-infection of TcI and TcIV. Blood meal analysis identified multiple feeding sources, including dogs (*Canis lupus*), humans (*Homo sapiens*), and wildlife species.

**Conclusions:**

This study provides insights into *T. cruzi* transmission dynamics in southern Texas, demonstrating the active role of domestic dogs and wildlife in the local cycle of infection suggesting endemism of *T. cruzi* in this region. These findings emphasize the need for continued surveillance and vector control measures to mitigate the risk of Chagas disease transmission in the USA.

**Supplementary Information:**

The online version contains supplementary material available at 10.1186/s13071-025-06917-6.

## Background

*Trypanosoma cruzi*, the etiological agent of Chagas disease, is primarily transmitted by triatomine insects. These insects act as vectors for the parasite, and their role in transmission is central to the epidemiology of Chagas disease, which affects millions of people across Latin America [1]. Although often described as an emerging disease in the USA, both *T. cruzi* and its triatomine vectors have been historically present in the southern regions of the country since at least the nineteenth century. The detection of *T. cruzi* DNA in a 1150-year-old Texan mummy further supports the long-standing presence of the parasite in the region [2–5]. However, only in recent years—and within the broader context of globalization— there have been increased interest and surveillance efforts in states such as Texas, Louisiana, Arizona, Florida, and California, where species such as *Triatoma sanguisuga* have been documented. This has raised concerns about the potential risk of human exposure to *T. cruzi* in these areas.

In the USA, Chagas disease is now recognized as an emerging public health concern but not as an endemic disease [6]. It is estimated that more than 300,000 people in the USA are living with Chagas disease, with the majority of cases originating from Latin America [7, 8]. Autochthonous cases of Chagas disease in the USA are extremely rare, with only 28 documented cases from 1955 to 2015 [9]. These are cases where the infection was likely acquired locally within the USA, rather than through travel to endemic areas or other means. Although human cases are relatively rare, the presence of domestic and wild reservoirs, along with vector species capable of transmitting the parasite (*T. sanguisuga, T. gerstaeckeri, T. protracta, T. rubida*, and *T. recurva*), increases the risk of zoonotic transmission. Dog kennels, as environments with domestic animal reservoirs, may serve as hotspots for the transmission of *T. cruzi* through triatomine vectors [10–12].

*Trypanosoma cruzi* exhibits significant genetic diversity in the USA [13]. The parasite is classified into discrete typing units (DTUs), with at least seven recognized: TcI-TcVI and TcBat [14, 15]. In the USA, TcI is the most prevalent DTU, accounting for approximately 42.4% of genotyped samples. This is followed by TcIV-USA, a North-American-specific lineage genetically distinct from South American TcIV, which represents around 25% of samples in some studies [13, 16]. *T. cruzi* has been detected in 18 states, with the highest concentration in Texas, Louisiana, and New Mexico [16]. The parasite’s genetic diversity in North America is high, with TcIV-USA showing significant divergence from other previously defined *T. cruzi* DTUs, suggesting a potential need for further investigation into its taxonomic positioning [17].

Studies on *T. cruzi* and triatomine vectors in dog kennels are limited but suggest that these environments can play a role in the transmission of *T. cruzi*. Triatomine species such as *T. sanguisuga*, found in Southern Texas, have been identified in rural areas, including dog kennels, where domestic dogs, known to harbor *T. cruzi*, may serve as reservoirs for the parasite [18–20]. The close proximity of dogs in kennels increases the likelihood of vector exposure, as triatomines can feed on infected dogs and potentially transmit the parasite to other animals or humans. Some studies have found *T. cruzi* DNA in triatomine vectors collected from these areas, indicating that transmission is occurring [12, 21, 22]. However, more comprehensive research is needed to understand the dynamics of *T. cruzi* transmission in dog kennels, the role of dogs as reservoirs, and the impact on public health, especially in regions where Chagas disease is emerging, such as in Southern Texas.

The aim of this study is to investigate *T. cruzi* infection in triatomine vectors collected from dog kennels in southern Texas by employing quantitative PCR for detection, next-generation sequencing (NGS) of the mini-exon gene for *T. cruzi* genotyping, and NGS of the 12S rRNA gene to identify the blood-feeding sources of triatomine vectors. By utilizing these molecular techniques, we aim to enhance the understanding of the transmission dynamics of *T. cruzi* in this region and provide insights into the role of domestic animals, particularly dogs, as key reservoirs of the parasite.

## Methods

### Sampling

**Fig 1.**
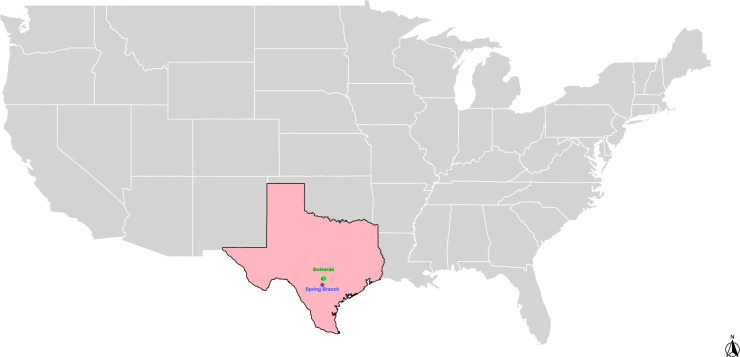
Geographical distribution of *Triatoma sanguisuga* collected in this study. The points indicate the locations in Texas where *T. sanguisuga* samples were collected

Sampling was conducted in Bulverde (29.7433° N, −98.4531° W and 29.780072° N, −98.395693° W) and Spring Branch (29.8878° N, −98.4267° W), Texas (Fig. 1), using hand collection and black light vane traps (BioQuip). A total of 48 adult triatomine insects were collected (Table S1). Hand collections were conducted at night during May and June 2023, using headlamps and tweezers to inspect dog kennels and other potential triatomine refuges. Light traps were set up at dusk and monitored for several hours to attract nocturnal insects (Fig. S1).

Taxonomic identification was performed in the laboratory under a stereoscope, following the morphological key by Lent and Wygodzinsky [21]. All specimens were identified as *Triatoma sanguisuga*. Only adult individuals were collected, and sex was determined during morphological identification, yielding 36 males and 12 females (Supplementary Table 1).

Following identification, each specimen was placed in a labeled Falcon tube containing ethanol and stored according to its collection site. This procedure ensured the preservation of DNA quality and minimized degradation prior to molecular analysis. Total sampling effort consisted of 27 h of manual searching per site, distributed over multiple nights.

### Molecular detection of *T. cruzi*

DNA extraction was performed using the entire abdominal section of each triatomine to ensure consistency and maximize the recovery of parasite DNA. Abdomens were placed into ZR BashingBead^™^ Lysis Tubes and subjected to mechanical disruption using a Disruptor Genie^®^ at 3000 rpm for 10 min. The resulting lysates were then incubated overnight at 56 °C with Proteinase K (Qiagen, Cat. No. 19131) and Buffer ATL (Tissue Lysis Buffer; Qiagen, Cat. No. 939011) to ensure thorough chemical lysis. Following lysis, samples were processed using an automated protocol on the chemagic^™^ 360 instrument with the Chemagic DNA Blood 400 Kit H96 (PerkinElmer), following the manufacturer’s instructions. This combined mechanical, enzymatic, and automated extraction approach was designed to ensure high-quality DNA suitable for downstream molecular applications [23–29]. For the detection and quantification of *T. cruzi* satellite DNA, we utilized a quantitative PCR (qPCR) protocol established by Velásquez-Ortiz et al. [26]. In this protocol, a TcI strain (MHOM/CO/04/MG) was used as the standard curve for qPCR, ensuring accurate quantification; 12 s subunit ribosomal gene of triatomines was used as internal amplification control under the conditions and primers previously described [30].

### *T. cruzi* genotyping and blood sources

Samples testing positive by qPCR for *T. cruzi* DNA were subsequently genotyped using conventional PCR targeting the spliced leader intergenic region (SL-IR) of the mini-exon gene. This gene region was selected as it is highly conserved among *T. cruzi* strains, allowing for reliable genotyping [26, 31]. The amplicons were subsequently sequenced using Oxford Nanopore Technologies (ONT).

Additionally, in all samples, a 215-bp fragment of the 12S rRNA gene was amplified to identify the host blood sources from which the insects had fed as reported elsewhere [23]. The resulting amplicons were sequenced using ONT. This sequencing approach allows for higher resolution in the identification of blood meal sources compared with conventional methods, thereby providing valuable insights into the ecological dynamics of vector–host interactions and helping to identify potential reservoir species.

All PCR amplicons (SL-IR region and the 12S rRNA gene) were visualized on a 1% agarose gel to confirm successful amplification. After confirming successful amplification, sequencing libraries were prepared independently for each marker—SL-IR (300–350 bp) and 12S (215 bp)—using the Ligation Sequencing Amplicons—Native Barcoding Kit (catalog no. SQK-NBD114.96, Oxford Nanopore Technologies). This separation aimed to avoid inter-marker competition during sequencing and improve accuracy in target identification.

Library preparation involved several steps, starting with end repair and A-tailing of the PCR products using the NEBNext End Repair/dA-Tailing Module. The DNA was then ligated with barcodes using the NEB Blunt/TA Ligase Master Mix and the PCR Barcoding Kit (EXP-NBD196), facilitating multiplex sequencing within each marker. The final step in the library preparation involved adapter ligation using the NEBNext Quick Ligation Module Kit. Additionally, the protocol includes washes with Short Fragment Buffer (SFB), which selectively retains fragments smaller than 3 kb and optimizes the retention of target amplicons.

After adapter ligation and final purification, each library was quantified, and the appropriate volume was calculated using the NEBioCalculator (https://nebiocalculator.neb.com/#!/dsdnaamt) to reach a final loading amount of 20 fmol. A total of 12 µL per library was loaded onto an R10.4.1 flow cell (FLO-MIN114) and sequenced on a MinION Mk1C device using MinKNOW software v24.02.16 (Oxford Nanopore Technologies), following the protocol recommended in the Ligation Sequencing Amplicons—Native Barcoding Kit 96 V14.

ONT was selected for its high-throughput capacity and flexibility to accommodate a range of amplicon sizes, enabling both the resolution of *T. cruzi* discrete typing units (DTUs) via SL-IR sequencing and the identification of vertebrate host species using shorter 12S mitochondrial fragments. To avoid competition between markers of different sizes and ensure data quality, sequencing runs for each target (SL-IR and 12S) were performed independently.

Basecalling was performed using Dorado v0.7.2, a software designed to convert raw signal data into nucleotide sequences. Reads with a quality score below 10 were filtered out, as these low-quality reads could lead to erroneous results. The resulting FASTQ files were assessed for overall quality using Nanostat (https://github.com/wdecoster/nanostat) [32], a tool that provides detailed quality statistics for long-read sequencing data. Further filtering of the reads on the basis of quality and size was performed using SeqKit (https://github.com/shenwei356/seqkit) [33], with a minimum mean quality score of 10 to ensure only high-quality sequences were retained for downstream analysis.

Taxonomic assignment was performed using Centrifuge (https://github.com/DaehwanKimLab/centrifuge) [34], a tool that provides fast and accurate taxonomic classification of metagenomic data. The taxonomic assignments were validated through BLASTn analysis, where the PCR-derived reads were compared with reference databases to ensure a sequence identity greater than 98%. This stringent threshold was set to confirm the accuracy of our identification of *T. cruzi* DTUs and the blood-feeding hosts.

To ensure the reliability of our amplicon-based NGS for the 12S rRNA gene, we included Genomic DNA-Rat Male BioChain as a positive control. As positive controls for SL-IR amplification, we included genomic DNA from *T. cruzi* reference strains corresponding to DTU TcI (MHOM/CO/04/MG) and DTU TcIV (CANIII cl1). For negative controls, we used RT-PCR Grade Water (Invitrogen**)**, which was rigorously tested for contamination by prokaryotic and eukaryotic genomic DNA using 16S rRNA and 18S rRNA assessments. Notably, negative controls showed no detectable amplicons or reads from human DNA. Additionally, we incorporated DNA from a *Rhodnius prolixus* colony exclusively fed on *Gallus gallus* blood to verify the detection of *Gallus gallus* reads, confirming its use as a feeding source in these insects.

To further investigate the genetic relationships between the *T. cruzi* strains and identify potential new variants, we reconstructed a phylogenetic tree using maximum likelihood (ML) with IQ-Tree 2 (http://www.iqtree.org) [35, 36], using the generated sequences in this study. This method was chosen for its robustness in handling large datasets and its ability to generate accurate phylogenetic trees. Node support was assessed using traditional bootstrap (1000 replicates), abayes, and SH-aLRT methods to ensure the reliability of the phylogenetic tree. Substitution models were chosen using the Bayesian Information Criterion (BIC) via ModelFinder (http://www.iqtree.org/ModelFinder/) [37] to select the most appropriate model for the sequence data. The final phylogenetic tree was visualized using iTol (doi: 10.1093/nar/gkae268) [38], a web-based tool for the interactive visualization of phylogenetic trees.

Statistical analyses were performed using R v.4.4.1 (https://cran.r-project.org/bin/windows/base/) [39, 40]. Frequency analyses were conducted to summarize the distribution of *T. cruzi* infection, insect sex, and blood meal sources. A Kruskal–Wallis test was applied to compare median parasite loads between geographic locations, followed by Dunn’s post hoc test with Bonferroni correction when appropriate. To account for the clustered nature of the data on the basis of global positioning system (GPS) coordinates, a linear mixed-effects model was implemented with city as a fixed effect and collection site as a random intercept. Additionally, Fisher’s exact test was used to evaluate the association between blood meal source and infection status. These tests were selected to ensure robust statistical interpretation while considering sample structure and potential confounders.

## Results

**Fig 2.**
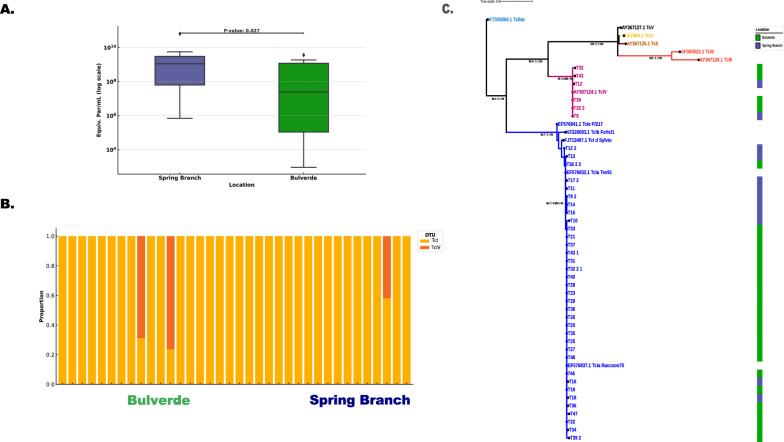
Parasite loads and genetic diversity of *Trypanosoma cruzi* from *Triatoma sanguisuga* samples. **A** Comparison of parasite burden in samples from Spring Branch and Bulverde. Values are expressed as equivalents of parasites per mL on a logarithmic scale. A significant difference was observed between the two locations (*p* = 0.027) **B** Proportion of *T. cruzi* discrete typing units (DTUs) detected in each sample included in this study **C** Phylogenetic tree inferred using maximum likelihood (ML) on the basis of *T. cruzi* mini-exon gene sequences. The analysis includes sequences from samples examined in this study and reference sequences from different DTUs of *T. cruzi* obtained from GenBank. Branch values represent bootstrap support, SH-aLRT, and Bayesian posterior probabilities, respectively. The scale bar indicates the number of substitutions per site

A total of 48 triatomine insects were collected from dog kennels in Bulverde (*N* = 37) and Spring Branch (*N* = 11), Texas. In Bulverde, 30 insects (81.1%) tested positive for *T. cruzi* PCR, while 7 insects (18.9%) were negative. All 11 samples from Spring Branch were positive for *T. cruzi* infection. The parasitic load was quantified in all positive samples, and the median parasitic load was log_10_ 8.09 equivalent parasites/mL (Fig. 2A; Table S1). To assess whether the parasitic load differed significantly between the two locations, a Kruskal–Wallis test was conducted. The results revealed a statistically significant difference in the median parasitic load between the locations (*H* = 4.87, *p* = 0.027). Dunn’s post hoc test with Bonferroni correction further indicated a significant difference between Spring Branch and Bulverde (*p* = 0.027), suggesting that the parasitic burden was higher in Spring Branch compared with Bulverde. In addition, to account for potential clustering by collection site, we implemented a linear mixed-effects model with city as a fixed effect and collection site (on the basis of GPS coordinates) as a random intercept. This model did not detect a statistically significant difference in parasite load between cities (*p* = 0.19), although moderate variability between collection sites was observed. These complementary analyses highlight that while parasite burden appears higher in Spring Branch, site-level variation must be considered in geographic comparisons.

The genotyping of *T. cruzi* PCR-positive samples using the mini-exon marker included 41 samples. Following trimming and quality filtering, we successfully recovered 19,475 reads from 37 samples, with an average of 500 reads per sample. The remaining four samples did not produce visible bands during electrophoresis and were therefore not subjected to sequencing due to insufficient amplicon yield. All samples were genotyped as *T. cruzi* TcI. Interestingly, two samples from Bulverde showed coinfection with TcI and TcIV, and one sample from Spring Branch also exhibited a TcI and TcIV coinfection (Fig. 2B). The phylogenetic reconstruction of the obtained mini-exon sequences confirmed that all TcI sequences clustered as the TcIa genotype, while TcIV sequences grouped with reference sequences of this DTU (Fig. 2C).

**Fig 3.**
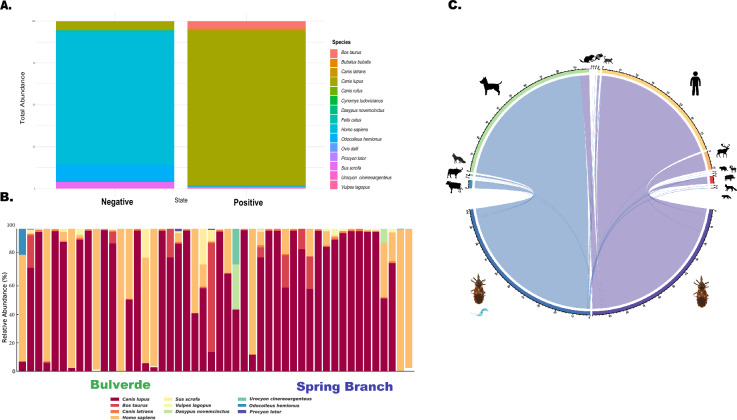
Feeding sources of *Triatoma sanguisuga* samples. **A** Proportion of feeding sources in *T. cruzi*-negative and *T. cruzi*-positive samples **B** Relative abundance of feeding source species in *T. sanguisuga* samples collected from Bulverde and Spring Branch, grouped by location. **C** Circos plot illustrating the primary feeding sources identified in *T. cruzi*-negative and *T. cruzi*-positive *T. sanguisuga* samples.

For the 12S rRNA gene, a total of approximately 5.377.457 reads were generated. After trimming and quality filtering, this was reduced to 4.557.167 reads, with an average of 94,000 reads per sample. After taxonomic assignment, a total of 32 unique blood-feeding sources were detected (Fig. 3A and B; Table S2). The most frequently identified hosts included *Canis lupus* (domestic dogs), *Homo sapiens* (humans), *Odocoileus hemionus* (mule deer), *Ovis* (sheep), and *Sus scrofa* (the genetic marker used does not allow for discrimination between domestic pigs and feral hogs). As a descriptive observation, most *T. cruzi*-positive samples had blood meals derived from domestic dogs (*Canis lupus*), whereas the few negative insects (*n* = 6) primarily fed on humans (*Homo sapiens*). However, Fisher’s exact test did not reveal a statistically significant association between host type (dog versus human) and infection status (*p* = 0.606) (Fig. 3C).

## Discussion

This study provides valuable insights into the transmission dynamics of *T. cruzi* in *T. sanguisuga* collected from dog kennels in southern Texas, contributing to our understanding of Chagas disease transmission in the USA, where the disease is “endemic” in specific regions [7, 41]. Our findings highlight the presence of *T. cruzi* infection in triatomine vectors in Texas, particularly in areas such as Bulverde and Spring Branch [21]. The significant parasitic load observed in these insects, with a median of log_10_ 8.09 equivalent parasites/mL, is consistent with previous reports of high parasitic burdens in triatomine vectors, which are critical in maintaining *T. cruzi* transmission cycles [42] (Fig. 2A). These findings likely suggest that an active transmission cycle of *T. cruzi* is occurring in the region.

The statistical difference in parasitic load between Bulverde and Spring Branch further suggests that ecological and biological factors may influence infection rates and the transmission potential of these vectors in the region. This has been suggested by some authors in different regions across the USA [19, 42]. The rural environment, larger property sizes, and greater outdoor access for dogs in Spring Branch may increase the likelihood of contact between vectors and infected hosts, facilitating more intense transmission cycles and higher feeding frequency in insects. In contrast, Bulverde is a more urbanized area, characterized by recent residential developments and limited interaction with wildlife or peridomestic reservoirs, which may account for the lower infection intensity observed. These differences in habitat structure and host availability likely contribute to the observed variation in parasite load. Furthermore, the prevalence of *T. cruzi* in shelter dogs in Texas has been reported to be as high as 8.8%, which is higher than previously thought and suggests a significant risk of transmission in the area [22, 41]. Although we were unable to test the dogs in the kennels at the time of insect collection, the high parasitic load detected in these insects suggests a significant risk of transmission. Future studies should include both serological and PCR testing of these dogs to better assess infection frequency and transmission dynamics, as has been done in studies across Latin America [2, 43, 44]. The wide distribution of *T. sanguisuga*, one of the most common triatomine species in the USA, from eastern Texas to other southern states, underscores the potential for Chagas disease transmission across a broad geographic range [19]. These findings emphasize the need for increased surveillance and control measures, particularly in high-risk areas of Texas, to mitigate the threat of Chagas disease transmission to both humans and animals [7, 43].

Recent studies have identified multiple *T. cruzi* lineages (TcI, TcII, TcIV, TcV, and TcVI) in dogs from the USA, indicating a diverse parasite population in the region [45]. This genetic diversity may impact parasite infectivity, reproduction, and differentiation in vectors, as well as influence host immune responses and disease progression [46]. The genotypic analysis of the *T. cruzi* sequences from this study revealed a predominance of the TcI DTU (Fig. 2B and C), which is indeed the most widespread genotype in both the USA and South America [7, 47]. However, the detection of coinfections with TcIV, particularly in samples from Bulverde and Spring Branch, aligns with findings from studies across the Americas where multiple *T. cruzi* genotypes have been observed [1, 15, 47]. In general, recent molecular typing studies in the USA have identified only two main genotypes: TcI and TcIV, with incipient reports of other DTUs such as TcV [48]. This is in contrast to South America, where six distinct genotypes (TcI-TcVI) have been found [3]. The limited diversity of *T. cruzi* genotypes in the USA may be due to fewer introductions of other genotypes or a lower diversity of natural reservoir hosts compared with South America [49], or even incomplete systematic sampling. The phylogenetic clustering of TcI strains as TcIa and the TcIV sequences matching reference strains from this DTU underscore the potential for diverse *T. cruzi* populations to coexist in the same vector population. However, most genotyping studies in the USA have relied on the mini-exon gene, which lacks sufficient phylogenetic resolution [50]. Future research should adopt multilocus approaches to accurately capture the genetic diversity of *T. cruzi* in the country and potentially identify novel DTUs, as previously reported [51].

The analysis of blood meal sources in *T. sanguisuga* revealed a diverse feeding pattern, underscoring its opportunistic behavior. The most frequently identified hosts were *Canis lupus*, *Homo sapiens*, *Odocoileus virginianus*, *Procyon lotor*, and *Didelphis virginiana*. Although most *T. cruzi*-positive insects had fed on domestic dogs and the negative ones on humans, statistical analysis using Fisher’s exact test did not show a significant association between host type and infection status (*p* = 0.606). This limitation highlights the need for future investigations with larger and more balanced sample sizes to better evaluate host-related transmission dynamics.

Nonetheless, the high proportion of *T. sanguisuga* specimens infected with *T. cruzi* that had fed on dogs reinforces the role of domestic canines as potential key reservoirs in the local transmission cycle. Some insects also exhibited mixed blood meals, suggesting multiple feeding events that may enhance parasite transmission (Fig. 3B and C). The detection of human DNA in several samples raises concern about vector–human contact and highlights the need for targeted surveillance to mitigate Chagas disease risk in endemic areas.

Blood meal source analysis provides valuable insights into the local transmission dynamics of *T. cruzi* and has been instrumental in studies across Latin America [28, 29, 52]. Domestic animals, particularly dogs, are often implicated as primary hosts facilitating *T. cruzi* transmission to humans, a pattern consistent with findings from other regions [53–55]. In Texas, recent studies have revealed higher-than-expected seroprevalence of *T. cruzi* in shelter dogs, with rates reaching up to 8.8% across multiple ecoregions [41], underscoring a substantial risk of domestic transmission. Our findings highlight the importance of adopting targeted surveillance and control approaches, such as the One Health framework for the prevention of Chagas disease in Texas, where *Triatoma sanguisuga*, domestic reservoirs, and human populations coexist within diverse ecological contexts. The detection of *T. cruzi*-infected vectors feeding on both domestic dogs and humans underscores the potential for sustained peridomestic and zoonotic transmission. Public health strategies in Texas should prioritize integrated vector management programs, including routine insecticide application in high-risk areas such as dog kennels, structural housing improvements to reduce vector entry, and community education initiatives to raise awareness of Chagas disease and its prevention. Additionally, systematic screening and treatment of domestic dogs as sentinels of transmission risk could enhance early detection and control efforts. These measures require coordinated action among public health, veterinary, and environmental sectors to effectively address the complex transmission dynamics of *T. cruzi* in Texas [1, 56].

The prevalence of blood meals from wild animals, such as mule deer and wild boar, reflects the complex transmission dynamics of *T. cruzi* in Texas. Blood meal analysis revealed a higher proportion of feedings on canines in Spring Branch, whereas human blood meals predominated in Bulverde. This pattern likely reflects differences in landscape structure and host availability. The rural setting and greater outdoor access for dogs in the Spring Branch may increase contact between vectors and canine hosts, promoting peridomestic transmission of *T. cruzi*. In contrast, the more urbanized environment of Bulverde, with higher human density, may explain the increased frequency of human blood meals. These ecological differences likely shape local transmission dynamics and should be considered when assessing Chagas disease risk. More than 100 known mammalian reservoirs, including domestic, peridomestic, and wildlife species, have been identified in the state [53]. This diverse range of potential reservoirs, including wood rats, raccoons, and wild canine species, contributes to the maintenance of sylvatic transmission cycles [42, 53, 57]. Understanding these complex relationships between vectors, domestic animals, and wildlife reservoirs is crucial for developing effective strategies to prevent *T. cruzi* transmission to humans in Texas. However, it is important to note that despite the presence of these risk factors, autochthonous human Chagas disease appears to be of relatively low risk in the USA [58]. This may be due to factors such as the inefficient stercorarian transmission of the parasite, the opportunistic feeding habits of triatomines on various vertebrate species, and the biological characteristics of the sylvatic triatomine species indigenous to the southern USA [22, 59]. Although the vectorial capacity of *Triatoma sanguisuga* has historically received less attention than other triatomine species, some studies have begun to elucidate its behavioral traits relevant to *T. cruzi* transmission [60, 61]. These findings reinforce the need for continued behavioral and ecological research to fully understand the epidemiological role of *T. sanguisuga*, particularly in the southern USA. In contrast, species such as *Rhodnius prolixus* and *Triatoma infestans* in Latin America have been extensively studied, providing critical insights into their role in *T. cruzi* transmission. This gap in knowledge highlights the urgent need for mechanistic and biological studies to elucidate the behavioral and physiological factors that influence *T. sanguisuga*’s ability to transmit *T. cruzi*. A deeper understanding of these aspects will be essential for developing effective vector control strategies and mitigating the risk of Chagas disease in the southern USA.

The situation in the USA differs significantly from South American countries in several ways. While South America has multiple triatomine species, the USA has 11 recognized species, with *T. sanguisuga* having the widest distribution across 23 states in the southeastern USA [18]. This species has been found infected with *T. cruzi* in Texas, Oklahoma, Louisiana, Alabama, Tennessee, Georgia, Delaware and Florida. In South American countries such as Bolivia, the seroprevalence of *T. cruzi* infection can be as high as 6.75%, while in the USA, estimates suggest a prevalence of about 1.24% among Latin-American-born residents in Los Angeles [62]. While South American countries report numerous locally acquired cases, only seven autochthonous vector-borne human infections have been reported in the USA, specifically in Texas, California, Tennessee, and Louisiana [63]. The ability to detect *T. cruzi* infection in both *T. sanguisuga* and wildlife reservoirs, combined with the high rate of domestic dog feeding, indicates that transmission cycles may be active in U.S. regions bordering Mexico and in areas with high rates of imported cases. For example, in Texas, where *T. gerstaeckeri* is prevalent, 57.7% of tested specimens were found to harbor *T. cruzi* [3]. This raises important concerns about the endemic nature of Chagas disease in the USA and its potential to become a growing public health threat if preventive measures are not implemented. The presence of autochthonous transmission, independent of migration, underscores the need for increased awareness, surveillance, and research on domestic vector populations and reservoir hosts. Unfortunately, historically the USA public health system has largely overlooked the threat posed by Chagas disease, further complicating efforts to address this emerging health issue. Recognizing and addressing the reality of locally acquired cases is crucial for developing effective public health strategies and preventing the further spread of *T. cruzi* within the country.

Despite the strengths of this study, several limitations should be acknowledged. First, the sample size was relatively small, which may limit the generalizability of our findings to broader triatomine populations in Texas. Future studies with larger sample sizes across multiple regions are needed to confirm these results. Second, while our molecular methods allowed for the detection and genotyping of *T. cruzi* and the identification of blood-feeding sources, the reliance on PCR and sequencing may introduce biases due to DNA degradation or amplification efficiency. Additionally, our study focused on adult triatomines, potentially overlooking the role of nymphs in transmission dynamics. The detection of mixed infections with TcI and TcIV highlights the complexity of *T. cruzi* transmission; however, our sequencing depth may have influenced the ability to detect low-abundance genotypes. Furthermore, bloodmeal analysis is limited by the persistence of host DNA in the insect gut, meaning recent meals are more likely to be detected, while older or partially digested meals may remain unidentified. Importantly, despite the high parasitic load observed in *T. sanguisuga* specimens, we were unable to detect *T. cruzi* infection in the dogs residing in the kennels where these insects were collected. This raises questions about alternative transmission dynamics and potential underestimation of canine infections due to limitations in sampling or diagnostic sensitivity. Finally, environmental and ecological factors influencing triatomine behavior and *T. cruzi* transmission, such as seasonal variations and host availability, were not assessed and should be considered in future research.

## Conclusion

our study also reinforces the importance of surveillance in domestic and wild animal populations as part of a comprehensive Chagas disease control strategy. The detection of *T. cruzi* in vectors feeding on both domestic and wild animals in Texas may serve as an early warning system for potential outbreaks and could inform future public health measures. Public health initiatives aimed at increasing awareness of Chagas disease, improving vector control, and implementing better diagnostic tools are essential to mitigating the risk of local transmission. This study lays the groundwork for further investigations into the ecological and genetic factors that drive *T. cruzi* transmission in the USA and supports ongoing efforts to better understand the role of triatomine vectors and animal reservoirs in Chagas disease dynamics across both North and South America.

## Supplementary Information

Supplementary material 1: Table S1. Metadata of *Triatoma* *sanguisuga* samples collected in this study.

Supplementary material 2: Table S2. Read counts for 12S rRNA gene.

## Data Availability

Sequence data that support the findings of this study have been deposited in the European Nucleotide Archive with the primary accession code PRJEB8664.

## Abbreviations

DTUs: Discrete typing units
ML: Maximum likelihood
qPCR: Quantitative polymerase chain reaction
PCR: Polymerase chain reaction
NGS: Next-generation sequencing
USA: United States
